# Medical Teachers’ Association

**Published:** 1860-07

**Authors:** 


THE CHICAGO
MEDICAL EXAMINER

VOL. I. JULY, 1860. No. 7

### ORIGINAL COMMUNICATIONS.

### MEDICAL TEACHER'S ASSOCIATION.

*New Haven*, June 4th, 1860.

The Convention of Medical Teachers, according to adjournment at Louisville, Ky., met at 10 o'clock, A. M., in the lecture room of Yale Medical College, Dr. Dixi Crosby, President, in the chair, and on motion, adjourned to meet again at 3½ o'clock, P. M.

AFTERNOON SESSION.

The Convention met according to adjournment, and was called to order by the President. The Secretary being absent, on motion of Prof. Palmer, of Mich., Prof. H. A. Johnson was elected Secretary.

The minutes of the Convention at Louisville, were read, and on motion, a list of the delegates was prepared, from which it appeared that the following institutions were represented: The Long Island College Hospital, by Prof. Austin Flint; Med. Department of Dartmouth College, by Prof. Dixi Crosby and O. P. Hubbard; Medical Department of the University of Louisville, Ky., by Prof. R. J. Breckenridge; Savannah Medical College of Georgia, by Prof. R. D. Arnold; Medical Department of Yale College Conn., by Profs. Jonathan Knight and Benj. Silliman, Jr; Medical Department of the University of Michigan, by Prof. A. B. Palmer; Harvard Medical College of Mass., by Profs. D. Humphreys Storer and G. C. Shattuck; Berkshire Medical College, of Mass., by Prof. W. H. H. Thayer; Medical College of Virginia, by Prof. J. B.

McCaw; Atlanta Medical College, of Georgia, by Prof. Jas. P. Logan; Missouri Medical College, by Prof. Jos. N. Mc Dowell; Medical Department of Lind University, Ill., by Profs. N. S. Davis and H. A. Johnson; Medical College of South Carolina, by Prof. Henry R. Frost; Iowa University, by Profs. D. L. McGugin and Daniel Meeker; Geneva Medical College, by Prof. Frederick Hyde; Albany Medical College, by Prof. Alden March.

The committee appointed at the previous convention to confer with a similar committee of the American Medical Association, reported through their chairman, Prof. Shattuck, a preamble giving an account of their doings, and proposing a series of resolutions, as follows:

1st. *Resolved*, That the Medical Colleges represented in this Convention, are willing to adopt the rule, if it be recommended by the American Medical Association, that every candidate for the degree of Doctor in Medicine must present certificates of having assiduously studied medicine during the period of three full years under the direction of a regular practitioner of medicine, recognized as such by the American Medical Association, who shall certify to the same under his own hand, and of attendance on two *full* courses of medical lectures in a medical school, recognized as regularly organized by the American Medical Association, with an interval of at least three months between the termination of the first course and the commencement of the last.

2d. *Resolved*, That the medical colleges represented in this Convention, are willing to keep a register of their students, in which shall be entered the name, the age, the period of commencing medical studies, and diploma already received, with the name of the college conferring it, and the name of the preceptor.

3d. *Resolved*, That the medical colleges represented in this convention, allowing that the proposed plan of admitting delegates from State Societies to attend the examination of the candidates for the degree of Doctor in Medicine to have been successfully carried out in several places do not think that it can with advantage be universally adopted; but, at the same time they are ready to ascertain and discuss any other measure by which the admission of unsuitable and unworthy members within the ranks of the profession can be prevented.

4th. *Resolved*, That this Convention earnestly recommend the American Medical Association to adopt such measures as will secure the efficient practical enforcement of the standard of preliminary education adopted at its first organization in May, 1847, or of a standard put forth by the medical society of the State in which a college is located, and, that medical colleges will thankfully receive and record the certificates alluded to in said standard, and one of moral character, whenever the profession generally, and the preceptors, will see that students are properly supplied with them.

5th. *Resolved*, That Hospital Clinical instruction constitutes a necessary part of medical education, and that every candidate for the degree of Doctor in Medicine, shall be required to have attended such instruction regularly for a period of not less than four months.

6th. *Resolved*, That the members of this Convention are ready to co operate in any efforts by which the attention of the community and of legislatures shall be called to the importance of the endowment of medical colleges and professorships.

7th. *Resolved*, That the attention of the American Medical Association be called to the proofs, in a letter from a German Medical Professor, of the degree of Doctor in medicine being conferred in Germany on unsuitable persons to be used in this country.

On motion of Prof. Davis, the report was received, and the resolutions taken up seriatum.

Prof. Flint moved to amend the first resolution by omitting the words "with at least an interval of three months between the termination of the first and the commencement of the last."

The amendment was discussed somewhat at length by Profs. Flint, McDowell, Davis, Palmer, Shattuck, Arnold, Frost and Logan, after which it was rejected.

On motion of Prof. McDowell, the first resolution was laid on the table, to be taken up at a future time.

On motion of Prof. Thayer, the second resolution was adopted.

The third resolution was discussed by Profs. McCaw, Breckenridge, Knight, Palmer, McDowell and Davis.

Prof. Logan offered the following as a substitute for the whole report:

*Whereas*, It is apparent that the Medical Colleges of the United States are not disposed to adopt the measures indicated by the American Medical Association, for the establishment of a higher system of medical education, as manifested by the failure upon the part of a large portion (and among the number some of the most prominent) to be represented at the Convention of Colleges. held last year in Louisville, and by a renewal of the same course of action towards the adjóurned meeting of said Convention, and as no action on the part of the Colleges represented would be likely to effect any change in the present system of medical education, and any attempt on the part of this limited representation to initiate any reform might be regarded as an offensive assumption of power, therefore,

*Resolved*, That this body declines to act for the Medical Colleges of the United States.

*Resolved*, That in the Medical Colleges alone resides the power ot effecting any desirable change in the present system of medical education, and it is only from their united action that any good result can be expected.

*Resolved*, That a Committee of—— be appointed to report the action of this body to the American Medical Association.

The substitute was discussed by Profs. Logan, Shattuck, Crosby, McGugin, McDowell, Storer and Palmer, and finally rejected.

At this stage of the proceedings Prof. Logan retired from the Convention, stating that he did not feel at liberty to act with it as the representative of the Atlanta Medical College.

On motion, the convention adjourned till Tuesday morning at 9 o'clock.

---

### SECOND DAY'S PROCEEDINGS.

June 5.

The Convention was called to order by the President, Dr. Crosby.

The following additional Institutions were represented: University of Maryland, by Prof. Edward Warren; University of Buffalo, by Prof. Thomas F. Rochester and Jas. P. White; St. Louis Medical College, by Prof. J. B. Johnson; Castleton Medical College, by Prof. E. K. Sanborn; Maine Medical College, by Prof. Nourse.

On motion of Prof. Shattuck the third resolution was adopted.

On motion of Prof. McDowell the fourth resolution was adopted.

On motion, the order of business was suspended, when Prof. Frost presented the following communication in regard to Medical Education in the South.

"I should wish to be heard while I make a few remarks on the progress of Education at the South, and the advances we have made in fulfilling the requirements of the Association. The report in my hand of the Dean of the Medical College of the State of South Carolina, of the graduates of that College and their requirements, presents a total of 114 graduates—all of whom had a preparatory education, such as the Association requires. Nearly all, with the exception of six, have had good literary opportunities: some graduates of colleges, others of academies of high repute, others instructed in the classics. Even those whose studies were confined to English, have had their minds strengthened by the study of Mathematics.

In making this statement, I would not be understood to say that they were well versed in the classics: but they have enjoyed the opportunity and profited in a greater or less degree by it. Neither would I be understood to say that our graduates are all Doctors. The diploma conferred is only an evidence that they have undergone a course of study; that they have been instructed in the principles of the profession, and made acquainted with the means by which they are to arrange and systematize the various occurrences presented to them—in short, that the foundation has only been laid by which they are to pursue advantageously their researches, and act for themselves. To be able Doctors and successful practitioners, requires years of study and observation, and there are many who after all this application have never been made Doctors.

The community in which a young graduate reside, soon becomes aware of this fact: it is only after a long apprenticeship, and years of toil and devotion to his business, that he acquires practice and confidence. Confidence is proverbially a plant of slow growth, and it is only after the individual has proved himself worthy that it is freely bestowed. Still, however, every Doctor has been a student, and as such has to endure taunts and imputations as to his qualifications. I well remember when a student in Medicine, forty-seven years since, fashionable ladies commented upon the homely appearance and neglected dress of the students of Philadelphia, and tauntingly observed that there was little to be observed in the streets but dogs and Virginia doctors! Yet from these classes of whom these remarks were made, there came forth a Wood, Mitchell, Meigs, McClelland, Hodge, Bartons, Darrach, and not to forget my own section, Dickson, Holbrook, Ramsay, and many others. Yet these young men were as ungainly as many at the present day; but they contained the gem as many of the present day, which required only to be polished. Education has been progressive to my observation; our graduates show their desire to excel by seeking opportunities abroad for greater requirements. In my day our reading was desultory and without system. My preceptor pointed to his library and told me to select my reading. My anatomical studies were pursued with a scalpel and the Dublin dissector. Our clinical instruction was nothing virtually. Mark the difference at the present time. Your winter and summer courses; your crowded hospitals; your private instructions, and your model plates, &c. All these speak trumpet-tongued that the work of improvement is onward."

On motion of Prof. McDowell it was directed to be appended to transactions of this body, for the American Medical Association.

On motion the fifth resolution was adopted.

On motion of Prof. McDowell the sixth resolution was adopted.

On motion of Prof. Shattuck, the seventh resolution was adopted.

On motion of Prof. Arnold the first resolution was taken from the table.

Prof. Shattuck offered for the first resolution a new one precisely the same as the first, with the exception of the last clause in regard to the interval of time between the first and last courses of lectures.

It was discussed by Prof. Shattuck, McDowell, Flint, Arnold, Breckenridge, Davis, Palmer, McCaw, Nourse and White.

Prof. White moved that the substitute and the original resolution be laid on the table. The motion was lost.

Prof. Breckenridge called for the vote on the substitute offered by Prof. Shattuck by colleges.

The substitute was lost by the following vote:

*Ayes*—Long Island College Hospital, Medical Department of Dartmouth College, Medical Department of the University of Michigan, Berkshire Medical College, Iowa University, Castleton Medical College, University of Buffalo, Maine Medical College—8.

*Noes.*—Medical Department of the University of Louisville, Savannah Medical College, Medical Department of Yale College, Harvard Medical College, Medical College of Virginia, Missouri Medical College, Medical Department of Lind University, Medical College of the State of South Carolina, Geneva Medical College, Albany Medical College, University of Maryland, St. Louis Medical College—12.

Profs. McGugin and Palmer, in voting for the substitute, explained that they did so because they were in favor of the propositions therein contained, and hoped that a distinct proposition, relating to the length of the *inter regnum* of courses similar to that contained in the original resolution might be presented, that they might vote for it.

The motion on the original resolution was then taken by colleges, and adopted by the following vote:

*Ayes*—Medical Department of Dartmouth College, Savannah Medical College, Harvard Medical College, Berkshire Medical College, Medical College of Virginia, Missouri Medical College, Medical Department of Lind University, Medical College of the State of South Carolina, Iowa University, Geneva Medical College, Albany Medical College, University of Maryland, St. Louis Medical College, Castleton Medical College—14

*Noes*—Long Island College Hospital, University of Buffalo, Maine Medical College—3.

On motion of Prof. Davis—

*Resolved*, That the Committee of which Dr. Shattuck is Chairman, be requested to report the doings of the Convention, with the resolutions adopted, to the American Medical Association.

On motion, the convention adjourned to meet again at the call of the President.

H. A. JOHNSON, Secretary.

